# Culture in embryonic kidney serum and xeno-free media as renal cell carcinoma and renal cell carcinoma cancer stem cells research model

**DOI:** 10.1007/s10616-017-0181-5

**Published:** 2018-02-17

**Authors:** Krzysztof M. Krawczyk, Damian Matak, Lukasz Szymanski, Cezary Szczylik, Camillo Porta, Anna M. Czarnecka

**Affiliations:** 1 Department of Oncology, Military Institute of Medicine, Szaserów 128, 04-141 Warsaw, Poland; 2 Present Address: Department of Translational Medicine, Center for Molecular Pathology, Skåne University Hospital, Lund University, Jan Waldenströms gata 59, 205-02 Malmö, Sweden; 3 School of Molecular Medicine, Warsaw Medical University, Ksiecia Trojdena 2a, 02-091 Warsaw, Poland; 4 Institute of Genetics and Biotechnology, Faculty of Biology, Warsaw University, Pawienskiego 5A, 02-106 Warsaw, Poland; 5 Present Address: Department of Microwave Safety, Military Institute of Hygiene and Epidemiology, 01-163 Warsaw, Poland; 6 Present Address: Medical University of Warsaw, Zwirki i Wigury 61, 00-001 Warsaw, Poland; 7 Medical Oncology, I.R.C.C.S. San Matteo University Hospital Foundation, Piazzale C. Golgi, 19, 27100 Pavia, Italy; 8 Italian Group of Onco-Nephrology - Gruppo Italiano di Onco-Nefrologia (G.I.O.N.), Pavia, Italy

**Keywords:** Renal cell cancer, Serum-free culture, Xeno-free culture, Cancer stem cells

## Abstract

The use of fetal bovine serum hinders obtaining reproducible experimental results and should also be removed in hormone and growth factor studies. In particular hormones found in FBS act globally on cancer cell physiology and influence transcriptome and metabolome. The aim of our study was to develop a renal carcinoma serum free culture model optimized for (embryonal) renal cells in order to select the best study model for downstream auto-, para- or endocrine research. Secondary aim was to verify renal carcinoma stem cell culture for this application. In the study, we have cultured renal cell carcinoma primary tumour cell line (786-0) as well as human kidney cancer stem cells in standard 2D monolayer cultures in Roswell Park Memorial Institute Medium or Dulbecco’s Modified Eagle’s Medium and Complete Human Kidney Cancer Stem Cell Medium, respectively. Serum-free, animal-component free Human Embryonic Kidney 293 media were tested. Our results revealed that xeno-free embryonal renal cells optimized culture media provide a useful tool in RCC cancer biology research and at the same time enable effective growth of RCC. We propose bio-mimic RCC cell culture model with specific serum-free and xeno-free medium that promote RCC cell viability.

## Introduction

Fetal bovine serum (FBS) is a component of high activity added to basic medium including RPMI (Roswell Park Memorial Institute medium), DMEM (Dulbecco’s Modified Eagle’s Medium), MEM (Minimum Essential Medium) or McCoy′s 5A or Ham’s F-12 media in order to promote cell growth and proliferation. FBS is in fact a mixture of multiple compounds and proteins. It is characterized by a high content of embryonic growth promoting factors along with many defined and undefined components—macromolecular proteins, low molecular weight nutrients, carrier proteins for water-insoluble components, hormones, attachment factors, spreading factors and low amount of gamma globulins (Shah 1999; Paschoal et al. 2014). Hormones carried by FBS include variable amounts of: insulin at a mean concentration of 10 µU/ml (range 6–14), cortisol, 0.5 ng/ml (0.1–23), growth hormone GH, 39.0 ng/ml (18.7–51.6), parathormone PTH, 1.72 ng/ml (0.085–6.18), triiodothyronine T3, 1.2 ng/ml (0.56–2.23), thyroxine T4, 0.12 ng/ml (0.08–0.16), thyroid-stimulating hormone TSH, 1.22 ng/ml (0.2–4.5), follicle-stimulating hormone FSH, 95 pg/ml (20–338), testosterone, 400 pg/ml (210–990), progesterone P4, 80 pg/ml (3–360), prolactin luteotropic hormone LTH, 176 pg/ml (20–500), luteinizing hormone LH, 8 pg/ml (1.2–18), prostaglandin E, 5.9 ng/ml (0.5–30.5), and prostaglandin F, 12.3 ng/ml (3.8–42.0). FBS contains also variable amounts of TGF-beta, vitamin A, vitamin E, cholesterol, histamine, integrins or selenite (Gstraunthaler and Lindl 2013). Moreover the FBS composition varies depending on where and when it was collected. It is widely accepted that composition of FBS directly influences the outcome of cellular experiments. It introduces the greatest variability into cell culture in experiment to experiment and between laboratories. FBS was therefore termed black box of cell culture (Marazzi et al. 2011). Relatively little is known about the impact of FBS hormones on a particular cell type and this further rises difficulties in analysis of hormone dependant processes and hormone induced gene expression in cells. The use of FBS presents obstacles to creating and publishing reproducible experimental results. In particular hormones act globally on cell physiology and influence systemic transcriptome and metabolome changes. Whereas hormone levels are typically tightly regulated in vivo, they are no longer subject to these regulations in vitro in FBS-based cell culture. FBS in bottles typically originates from both sexes and therefore the combination of testosterone and estrogen in a single batch of serum remains uncontrolled (Kamei 1987; Zheng et al. 2006). Moreover interstitial space vary from organ to organ and its composition evolved to maintain location-specific conditions that are especially suited for the optimal survival and function of cells building a particular organ. Therefore, using FBS to culture specific cells in vitro is not only non-physiological but also may lead to aberrant results in any experiment because organ-specificity is not kept (Maggs et al. 1995; Bhave and Neilson 2011).

Renal cell cancer (RCC) was shown to be depended among other hormones on T3 and insulin stimulation (Solarek et al. 2015; Czarnecka et al. 2016). As a renal proximal tubule originating structure its interstitial space fluid composition is specific (Zeisberg and Kalluri 2015) and in the tumour microenvironment selected proteins, growth factors and hormones are secreted (Teng et al. 2011; Solarek et al. 2015; Tracz et al. 2016). Recently there is an increase in the development of cell culture media without serum supplementation in order to reduce the introduction of any undefined exogenous factor or infectious agent into experimental models. In the light of this FBS-raised challenges it is essential to develop more physiological alternative cell culture protocols for the culture of RCC cells in serum-free, animal component-free, and/or protein-free medium conditions enabling endocrine oncology research in bio-mimic RCC models.

Serum free media (SFM) do not contain supplementation with serum (including FBS), but may contain discrete amounts of proteins or bulk protein fractions. Protein free media (PFM) contain no proteins, but still may contain plant or yeast hydrolysates, which are non-physiological for human cells. Many of PFM are animal factors origin free. Finally chemically defined media (CDM) contain no proteins, hydrolysates, or components of unknown composition or structure. These media are animal origin free and all components have a defined, known chemical structure without addition of peptides, hormones, growth factors which are found with typical serum-based culture method (Fike et al. 2001; van der Valk et al. 2010). Completely defined biomimic cell culture systems eliminate experimental variability, improve intra- and inter-laboratory reproducibility, decrease possibility of contamination toxins and pathogens and save time on data analysis time (van der Valk et al. 2010).

For many cancer cells, insulin alone is sufficient to promote survival and proliferation under serum-free conditions. Most often normal or immortalized cultured human cells enter quiescence G_0_ under the same limited conditions and actually need few additional growth factors to proliferate (Pause et al. 1998). For Human Embryonic Kidney 293 (HEK293)—widely used renal cells—it was shown that serum-free medium that contains all the amino acids, l-glutamine, vitamins, 25 mM HEPES and insulin is needed for maximum growth without the need for transferrin supplementation (Cervera et al. 2011). Our aim was to study RCC cells in serum free media optimized for renal cells in order to select the best study model for endocrine research. Stem cell culture conditions were also tested. Thus, we assessed cell phenotype and growth kinetics in the cell culture media tested.

## Materials and methods

### Cell culture

Human kidney cancer stem cells (HKCSC, CD133 +) were obtained from Celprogen (Torrance, CA, USA) and have been cultured in Complete Human Kidney Cancer Stem Cell Medium (M36117-44S; Celprogen Inc., Torrance, CA, USA) as per manufacture protocol requirements—as positive control (optimal growth). 786-0 (CRL-1932)—primary tumour derived VHL mutated renal cell carcinoma cell line—was obtained from ATCC Global Bioresource Center (Manassas, VA, USA). As per ATCC protocol the base medium for 786-0 cell line Roswell Park Memorial Institute (RPMI)—1640 Medium with 2000 mg/l glucose and 25 mM HEPES (Cytogen, Lodz, Poland) was used. RPMI 1640 was supplemented with l-glutamine (Biochrom GmbH, Berlin, Germany) at 2 mM final concentration.

As we investigated also cells in hypoxia and glucose dosage influences the induction of extracellular and intracellular hypoxia we also used Dulbecco’s High Glucose Modified Eagles Medium (DMEM HG) with 4500 mg/l (25 mM) of glucose—as additional positive control (HyClone, GE Healthcare Life Sciences, Logan, UT, USA). DMEM was used since glucose uptake and lactate production are expected higher in hypoxia than in normoxia, oxidative phosphorylation is inhibited in high glucose concentrations by Crabtree effect and may regulate oxygen-dependent biological functions (Prior et al. 2014). DMEM was selected as it was used initially for the culture of mouse embryonic stem cells (Evans and Kaufman 1981) and therefore is expected to support growth of HKCSC.

RPMI 1640 Medium contains biotin, vitamin B_12_, and PABA, which are not found in DMEM. DMEM has a higher concentration in calcium (1.8 mM) and a lower concentration in phosphate (1 mM) than RPMI 1640 which contains 0.8 mM of calcium and 5 mM of phosphate. RPMI 1640 contains inositol and choline at very high concentrations. RPMI 1640 medium contains also sodium pyruvate and HEPES. RPMI 1640 uses a sodium bicarbonate buffer system (1.5 g/L) and therefore requires a 5–7% CO_2_ environment to maintain physiological pH, while DMEM uses a sodium bicarbonate buffer (3.7 g/L), and therefore requires a 5–10% CO_2_ environment to maintain physiological pH of cells, so 5% CO_2_ was used for both media.

Both RPMI 1640 and DMEM HG were supplemented with 10% FBS (Hyclone, GE Healthcare Life Sciences, Logan, UT, USA) and Pen-Strep (Penicillin–Streptomycin) solution (Sigma-Aldrich, St. Louis, MO, USA) at a final concentration of 100 IU/mL penicillin and 100 μg/mL streptomycin. Cells were cultured in 75 cm^2^ cell culture flasks (Orange Scientific, Braine-l’Alleud, Belgium).

Cells were cultured per good practice mammalian tissue culture protocols using sterile technique in a normoxic (21% O_2_; 5% CO_2_; 37 °C) incubator (Forma Scientific 3131; Thermo Fisher Scientific, Waltham, MA, USA) and a hypoxic (2% O_2_; 5% CO_2_; 37 °C) incubator (SANYO MCO-5 M; Moriguchi, Osaka Prefecture, Japan) (Geraghty et al. 2014).

### Xeno-free medium culture

Four serum free media—*EX*-*CELL 293* (Sigma-Aldrich), *IS 293*-*V™* (Irvine Scientific, Santa Ana, CA USA), *FreeStyle™ 293* (Gibco™, Thermo Fisher Scientific, Waltham, MA, USA)*, NutriStem XF/FF Culture Medium [NutriStem hPSC XF Medium]* (Stemgent, Cambridge, MA, USA) were tested.

*EX*-*CELL 293—***an animal-protein free, serum-free** medium developed for the long-term growth of HEK 293 and related cells, in a suspension culture without refeeding, for about 10 days and supporting cell growth for more than 20 passages with no loss of viability (Buckler and Al-Rubeai 2009).

*IS 293*-*V™—***a serum-free, animal-component free** medium without l-glutamine for suspension culture of HEK 293 cells for long term, high density cell growth with high levels of recombinant protein production is designed for use in 5% CO_2_.

*FreeStyle™ 293 expression medium*—**an animal origin**-**free, chemically defined, serum-free**, protein-free medium specifically developed to support the growth and transfection of 293-F cells in suspension containing GlutaMAX™ supplement.

*NutriStem XF/FF Culture Medium [NutriStem hPSC XF Medium]*—a fully defined **xeno-free**, **animal**-**component free**, **low growth factor** human embryonic stem (ES) and induced pluripotent stem (iPS) cell feeder-free culture medium enabling maintenance and expansion of pluripotent stem cells for at least 20 passages with supporting pluripotency marker expression, fast proliferation and stable karyotype and differentiation potential with low basic FGF (4 ng/ml), and TGFβ (< 5 ng/ml) and low-protein and stable L-alanyl-l-glutamine and HAS (Human Serum Albumin).

Human Kidney Cancer Stem Cell Complete Growth Medium Serum Free (HKCSC SF) (M36117-44) (Celprogem Torrance, CA, USA) was used as control of HKCSC serum free growth. For 786-0 cells RPMI 1640 (Cytogen, Lodz, Poland) without FBS was used as a control of serum free growth.

For cell morphology analysis xeno-free culture cells have been cultured for the first 24 h (day 1) after seeding in control DMEM HG medium to attach, and subsequent observation was conducted as follows 48 h (day 2) 100%—control medium, 72 h (day 3)– control medium: test medium 1:1; 96 h (day 4)—control medium: test medium 1:2; 120 h (day 5) and further—100% test serum free medium.

For growth curve analysis after thawing cells were grown till 100% confluence in the dedicated media—RPMI 1640 with 10% FBS for 786-0 and HKCSC compete medium with serum for HKCSCs. At full confluence cells were trypsinized and seeded in 96-well plates (day 0). Cells were left to attach for 24 h and measurements were started on the subsequent day (day 1).

### MTT test

Vybrant^®^ MTT cell proliferation assay kit (Thermo Fisher Scientific) was used as a method for determination of viable cell metabolism, since the signal read-out generated is dependent on the number of viable cells and their metabolic activity. Measurement was performed using microplate reader (Thermo Fisher Scientific) with absorbance recorded at 570 nm. 3-[4,5-dimethylthiazol-2-yl]-2,5-diphenyl tetrazolium bromide was used to asses cell metabolic activity of NAD(P)H-dependent cellular oxidoreductase enzymes, to reflect the number of viable cells (Hansen et al. 1989). The test was used to provide linearity of results up to 10^6^ cells per well. Since culture medium at elevated pH may cause an accelerated spontaneous reduction of tetrazolium salts (increased background) we also measured absorbance values from control wells without cells as a control (Riss et al. 2004). Final incubation with the MTT was performed after exchanging the cells into medium free of phenol red—DMEM, high glucose, HEPES, without phenol red (Gibco™, Thermo Fisher Scientific) for high glucose experiments and RPMI 1640 Medium, without phenol red (Gibco™, Thermo Fisher Scientific) for other read-outs.

### Alamar blue test

Alamar Blue was used to monitor the reducing environment of the living cell and for monitoring RCC cell proliferation and function—for the determination of the kinetics of cell growth (Rampersad 2012). Alamar blue measurements were performed every 24 h for 6 days with Multiskan™ GO microplate spectrophotometer (Thermo Fisher Scientific) at 570 and 600 nm according to the manufacturer’s protocol. Fluorescent signal from Alamar Blue was analysed as proportional to the number of viable cells and linear relationship between cell number and fluorescence was calculated. Resazurin is reduced by mitochondrial cytosolic and microsomal enzymes (Gonzalez and Tarloff 2001). The absorbance obtained from readings was recalculated to percentage reduction of Alamar Blue.

### Trypan blue (dye exclusion) test

Cell viability was determined with trypan blue (Sigma-Aldrich) exclusion method and manual counting using an haemocytometer—Bürker–Türk grid. The dye exclusion test was interpreted as for viable cells not taking up impermeable dye—remain colourless, and dead cells being permeable and taking up the dye—become blue (Strober 2001, 2015). Cell viability was calculated as the number of viable cells divided by the total number of cells within the grid on the hemacytometer: % viable cells = [1.00 − (Number of blue cells ÷ Number of total cells)] × 100. Trypan was used to stain cells with a damaged cell membrane (necrotic and apoptotic cells), since the trypan blue staining is negatively charged and does not interacting with cells unless the membrane is damaged (Sarma et al. 2000).

### Hormone testing in SF-media

For hormonal stimulation FreeStyle Medium supplemented with 3,3′,5-triiodo-L-thyronine sodium salt (Sigma-Aldrich, St. Louis, MO, USA) was used—1 pM T3, 4 pM T3, 1 nM T3 or 100 nM T3. TSH, T3, T4 depleted serum (SF231-2, BBI Solutions, Cardiff, UK) was used as control. To block the receptor—CAS 251310-57-3—thyroid hormone receptor antagonist 1-850 (Santa Cruz Biotechnology Inc., Dallas, TX, USA) was used at 1.5 μM inhibitor concentration. RCC cell proliferation inhibition was obtained with sunitinib malate (Sigma-Aldrich) as negative control. It was dissolved in DMSO and used at a final concentration of 3 µM. Statistical comparison between conditions was evaluated with GraphPad Prism 6 (GraphPad Software, Inc., La Jolla, CA, USA) with two-way ANOVA followed with Dunnett’s multiple comparisons tests—as described before (Curran et al. 2010; Czarnecka et al. 2016).

### Hanging drop 3D culture

For generation of tissue-like cellular aggregates hanging drop culture was utilized with 1000 cells seeded in 20 µl drop of DMEM medium spotted on the inner side of a 100 mm cell culture dish top. Top covered plate filled with 10 ml PBS for humidity support (Foty 2011). Nikon TMS Inverted Microscope (Nikon Instruments, Inc., Melville, NY, USA) with HDCE-30C 3MP camera (Delta Optical, Nowe Osiny, Poland) was used to visualize cells.

## Results

### Oxygen tension modulate RCC cell viability in different media

**Fig. 1:**
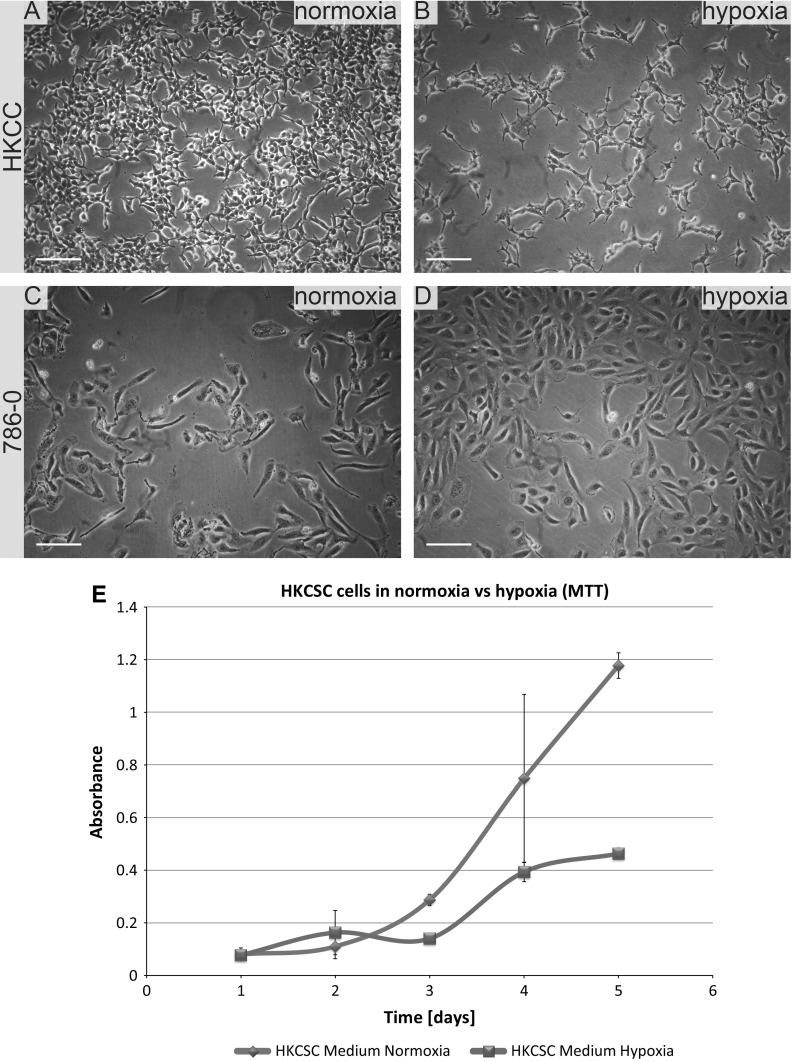

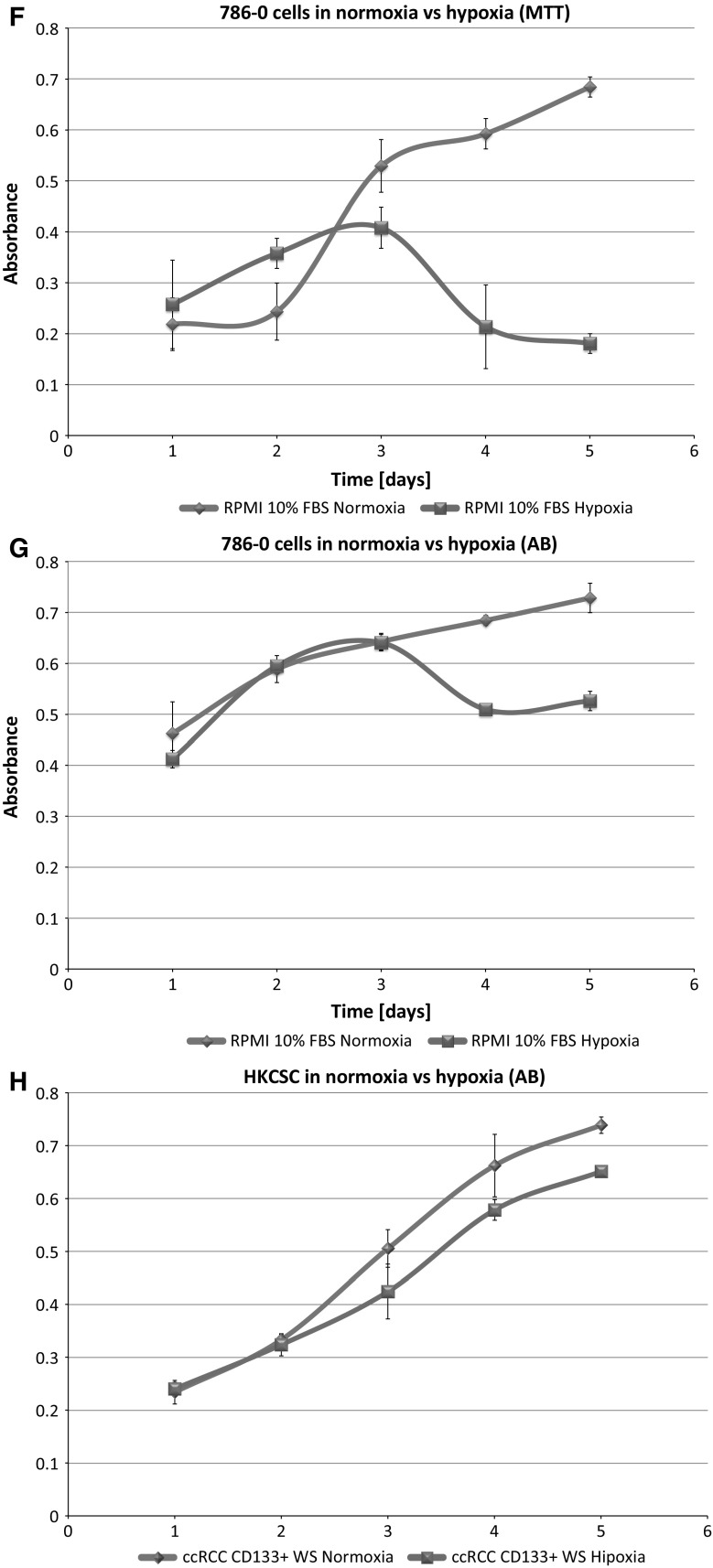

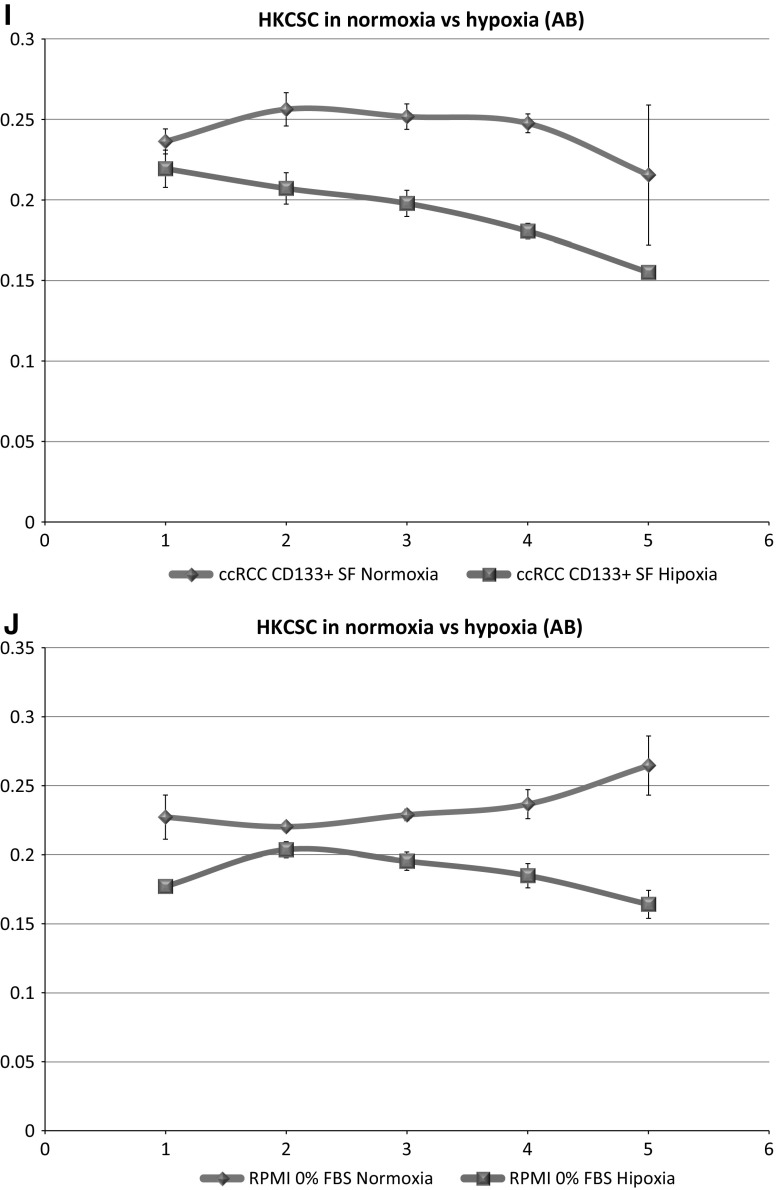
RCC cells morphology and growth in hypoxia and normoxia in control media. HKCSCs morphology in DMEM HG medium in **a** normoxia **b** hypoxia; 786-0 cells morphology in DMEM HG medium in **c** normoxia and **d** hypoxia; MTT-test of HKCSC cells in hypoxia and normoxia in HKCSC medium (**e**) and 786-0 cells in hypoxia and normoxia in RPMI 10% FBS medium (**f)**; Alamar Blue (AB)-test of 786-0 cells in hypoxia and normoxia in RPMI 10% FBS medium **g** HKCSC in hypoxia and normoxia in HKCSC medium (**h**), HKCSC in hypoxia and normoxia in HKCSC serum free medium (**i**) and HKCSC in hypoxia and normoxia in serum free RPMI medium (**j**). Scale bar = 100 μm

**Fig. 2:**
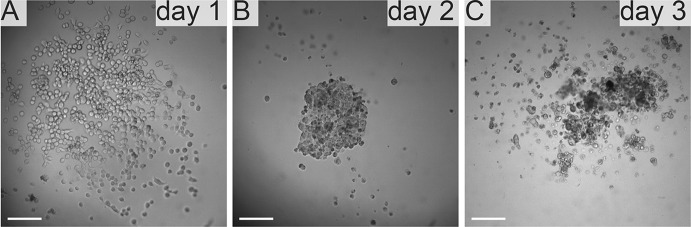
Human kidney cancer stem cells adhesion pattern. Hanging drop culture of HKCSCs in DMEM high glucose 10% FBS. 1000 cells per drop were seeded on the first day. Cells were cultured in the drop for 24 h and subsequently transferred to a 96 well conical bottom microwell plates. Presented is aggeation of HKCSC cells during culture: cells seeded are shown **a** after 24 h of culture, **b** after 48 h, and **c** after 72 h. Scale bar = 100 μm

In standard 2D cell culture in regular T-75 flasks we have observed that HKCSC cells proliferated faster in normoxia (Fig. 1a, b, e), while 786-0 cells showed higher proliferation rate in hypoxia over first 3 days and then proliferation rate decreased (Fig. 1c, d, f, g), as defined both by MTT and Alamar Blue reduction. Both HKCSC and 786-0 cells in DMEM and RPMI 1640 had similar cell growth curve—cell number (absorbance) value increased gradually from day 1 to day 3, significantly increased on day 4 and reached a peak on day 6 in normoxia. In hypoxia 786-0 cells on day 4 first decreased in viable cell number (Fig. 1f, g), while HKCSC were still viable both in HKCSCs dedicated medium (Fig. 1e—MTT, Fig. 1h—Alamar), but not in HKCSCs medium without serum (Fig. 1i) or serum (FBS) free control medium (Fig. 1j, Table 1). In DMEM-HG 786-0 cells exhibited a typical bi-polar epithelial cell-like morphology, while HKCSC are polygonal to spindle shaped. HKCSC harbours also a potential to form aggregates in hanging drop culture. In hanging drop 3D culture HKCSC initially spread homogeneously while seeding and no aggregates were formed on day 1. Solid and homogenous aggregates were formed on the 2nd day of culture (Fig. 2), but from the third day they started disintegrating. To investigate whether these differences between 786-0 cells and HKCSC might be caused by not optimal culturing conditions, and are not only cell-line specific, we systematically assessed available defined HEK media as described below.

**Table 1:** Renal cell cancer cell viability in selected media

Medium	786-0 cells	HKCSC cells
RPMI 1640 + 10% FBS	+++	+++
RPMI 1640 serum free	−	−
DMEM HG + 10% FBS	+++	+++
DMEM HG serum free	−	−
HKCSC with serum	++	+++ 2D and 10% 3D growth
HKCSC serum free	−	+ 2D and 3D growth
IS293 medium	−	−
ExCell medium	−	−
NutriStem medium	50% suspension growth in hypoxia	Overgrow in multiple layers in normoxia
	No cell detachment in normoxia	Form clusters in hypoxia
FreeStyle medium	+++	3D suspension growth

### SF-media supported RCC cell growth

**Fig. 3:**
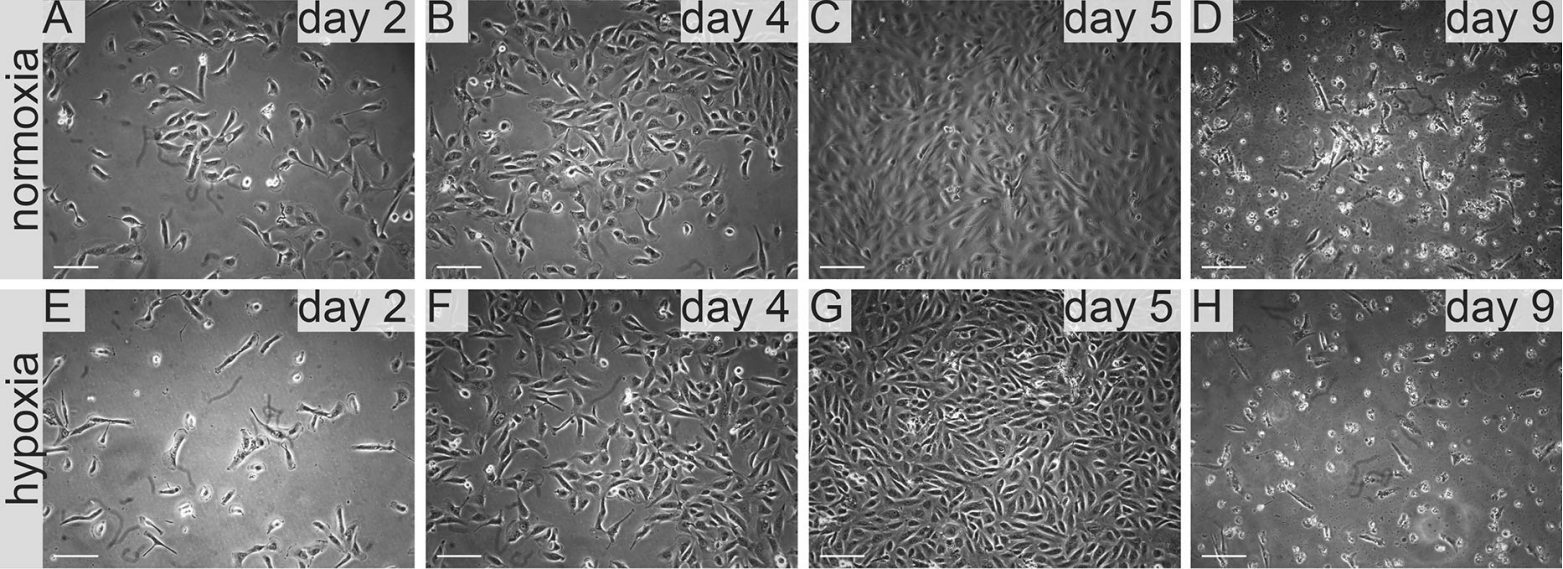
786-0 RCC primary tumour cell growth in IS293 medium in normoxia and hypoxia. Representative images present 786-0 cells cultured in IS293 medium in normoxia on **a** day two, **b** day four, **c** day five, **d** day nine; and in hypoxia, on **e** day two, **f** day four, **g** day five, **h** day nine. Scale bar = 100 μm

**Fig. 4:**
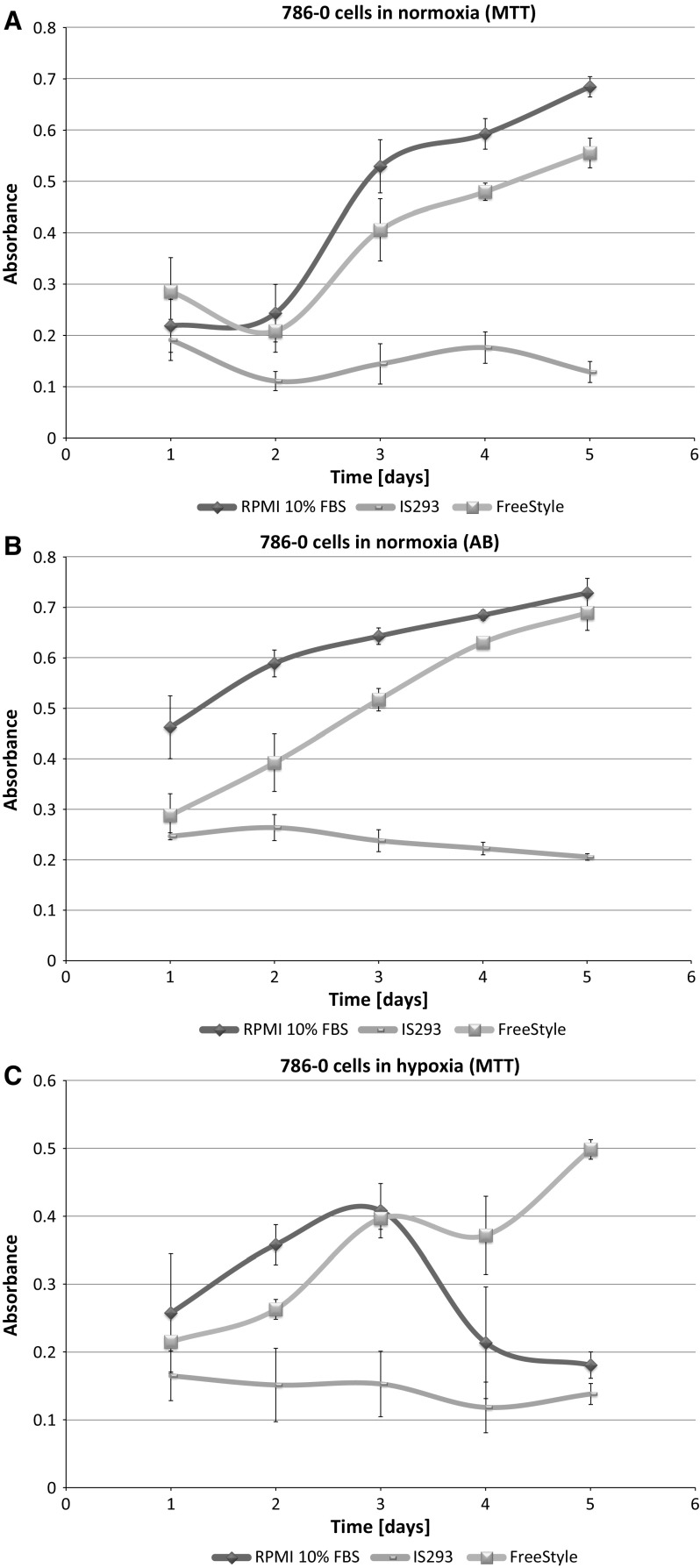

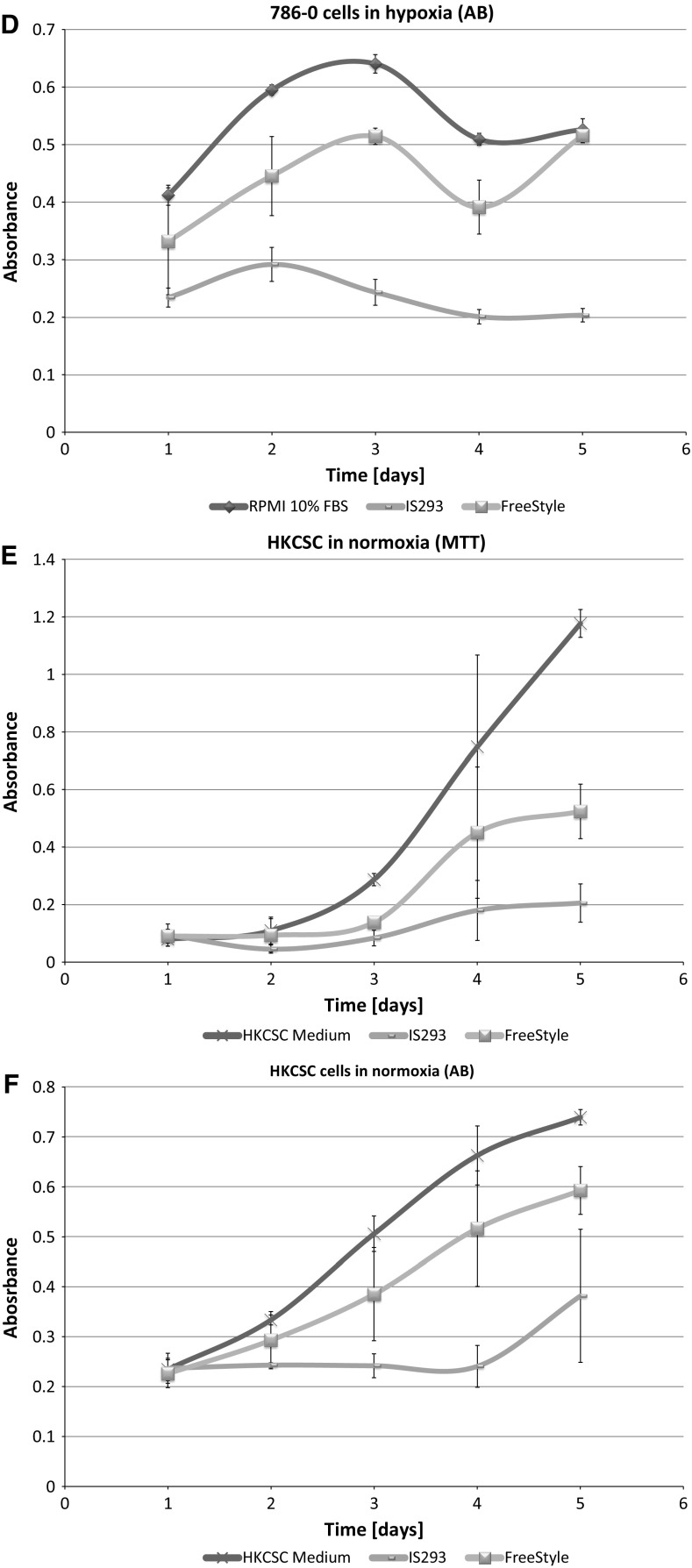

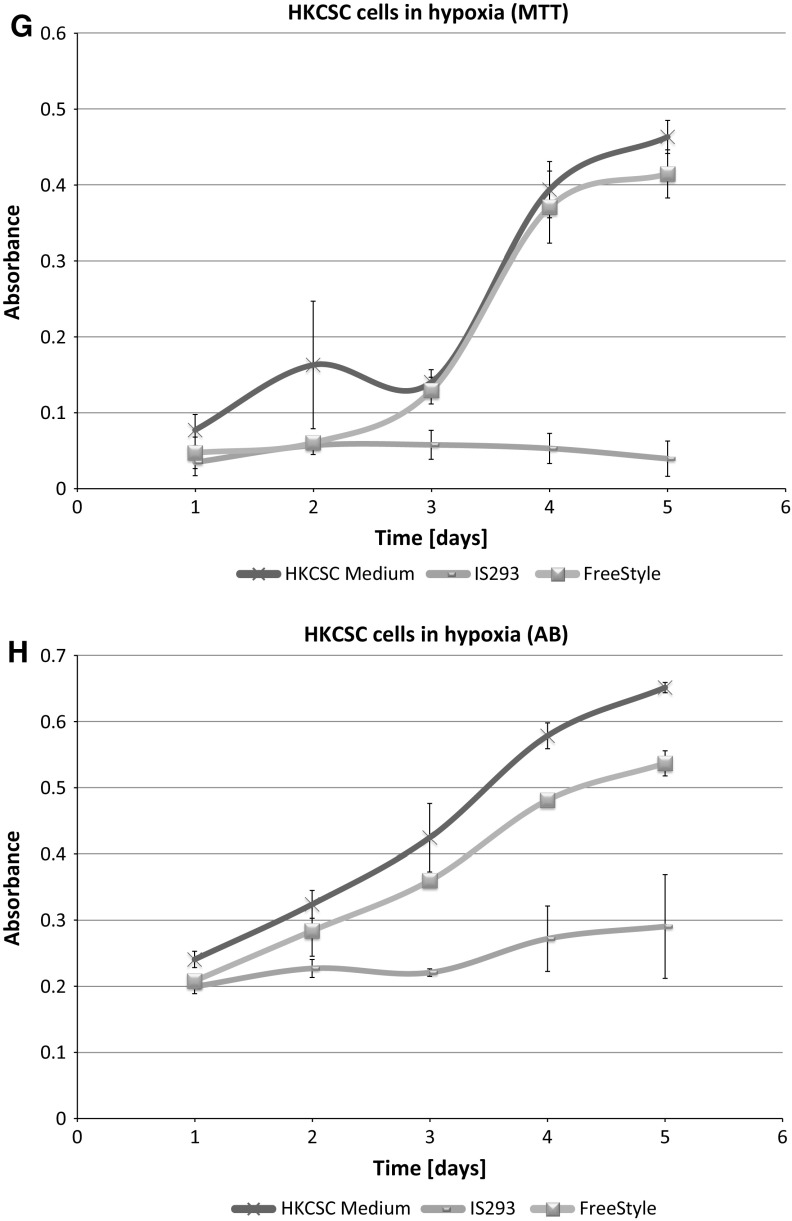
Renal cancer cells viability in the test media. 786-0 cells viability in RPMI + 10% FBS medium compared with culture in FreeStyle and IS293 media in normoxia as defined by MTT (**a**) and Alamar Blue (AB) **b** test; and in hypoxia as defined by MTT (**c**) and Alamar Blue (AB) (**d**) test. HKCSC cells viability in HKCSC medium with serum was compared with cultures in FreeStyle and IS293 media in normoxia as determined by MTT (**e**) and Alamar Blue (AB) **f** test; and in hypoxia as determined by MTT (**g**) and Alamar Blue (AB) **h** test

**IS293 medium** alone did not promote survival of RCC cells and marginally supported RCC stem cells viability and proliferation. We observed rapid cell proliferation and normal morphology in both normoxia and hypoxia during first days of the culture, as long as residual DMEM-FBS was present in culture. Culture in 100% IS293 medium caused cells to detach and die in both normoxia and hypoxia, with low number of surviving cells in normoxia. In normoxia 786-0 proliferated rapidly in 50–25% IS 293 medium, but in 100% cells detached rapidly and underwent cell death. In hypoxia 786-0 proliferated normally in 50–25% IS 293 medium, but in 100% cells detached and underwent cell death (Table 1). Low number of cells (up to 20%) detached in the first day of culture in hypoxia and subsequently after the next day of culture all cells detached. This was less intensive than in normoxia (Fig. 3). 786-0 cells did not proliferate in 100% IS293 medium, neither in normoxia (Fig. 4a, b), nor in hypoxia (Fig. 4c, d).

**Fig. 5:**
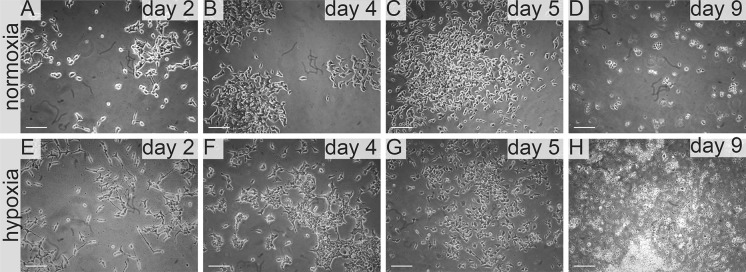
Human kidney cancer stem cell growth in IS293 medium in normoxia and hypoxia. Representative images present HKCC cells cultured in IS293 medium in normoxia on **a** day two, **b** day four, **c** day five, **d** day nine; and in hypoxia, on **e** day two, **f** day four, **g** day five, **h** day nine. Scale bar = 100 μm

HKCSCs proliferated in normoxia in IS23 supplemented medium, but at 100% concentration detached. In hypoxia HKCSCs proliferated generally more slowly than in normoxia. After 100% IS293 medium stimulation, HKCSCs displayed shape changes including cell dispersion and trapezoidal cell shape. Moreover on day 5 cells started to undergo cell death and were 100% dead on day 9 (Fig. 5). Low number of HKCSC cells proliferated in IS293 medium after day 4 in normoxia (Fig. 4e, f), but not in hypoxia (Fig. 4g, h).

**Fig. 6:**
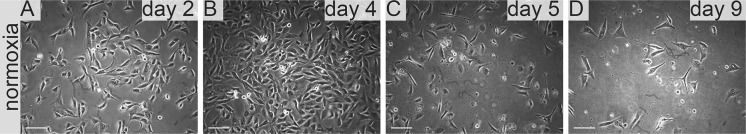
786-0 RCC primary tumour cells growth in ExCell medium in normoxia and hypoxia. 786-0 cells cultured in ExCell medium in normoxia on **a** day two, **b** day four, **c** day five, **d** day nine. Scale bar = 100 μm

Furthermore, another tested medium **ExCell medium** supported RCC growth only when supplemented with DMEM-FBS. In normoxia 786-0 proliferated rapidly in 50–25% ExCell medium, but in 100% cells detached and underwent cell death. We observed that cells proliferated rapidly and showed normal morphology in 25–50% ExCell medium. However, 100% ExCell medium caused cells to detach and to undergo cell death (Fig. 6, Table 1).

**Fig. 7:**
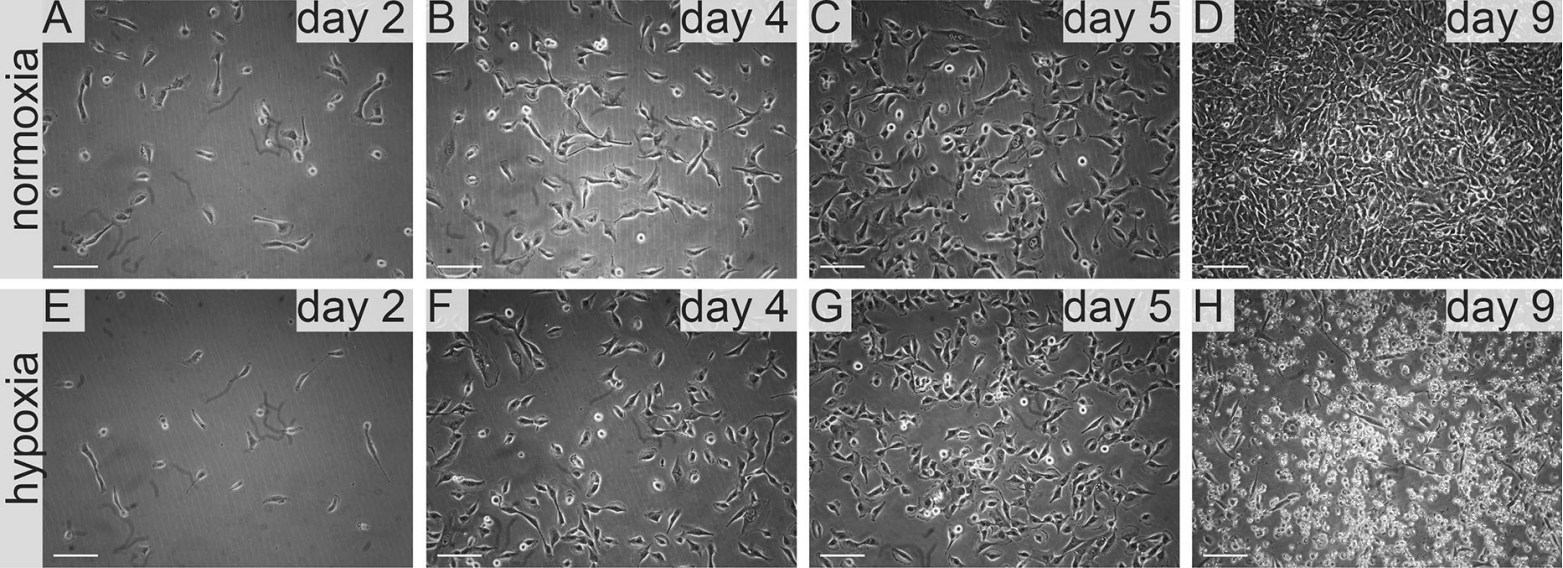
786-0 RCC primary tumour cell growth in NutriStem medium in normoxia and hypoxia. Representative images present 786-0 cells cultured in NutriStem medium in normoxia on **a** day two, **b** day four, **c** day five, **d** day nine; and in hypoxia, on **e** day two, **f** day four, **g** day five, **h** day nine. Scale bar = 100 μm

**Fig. 8:**
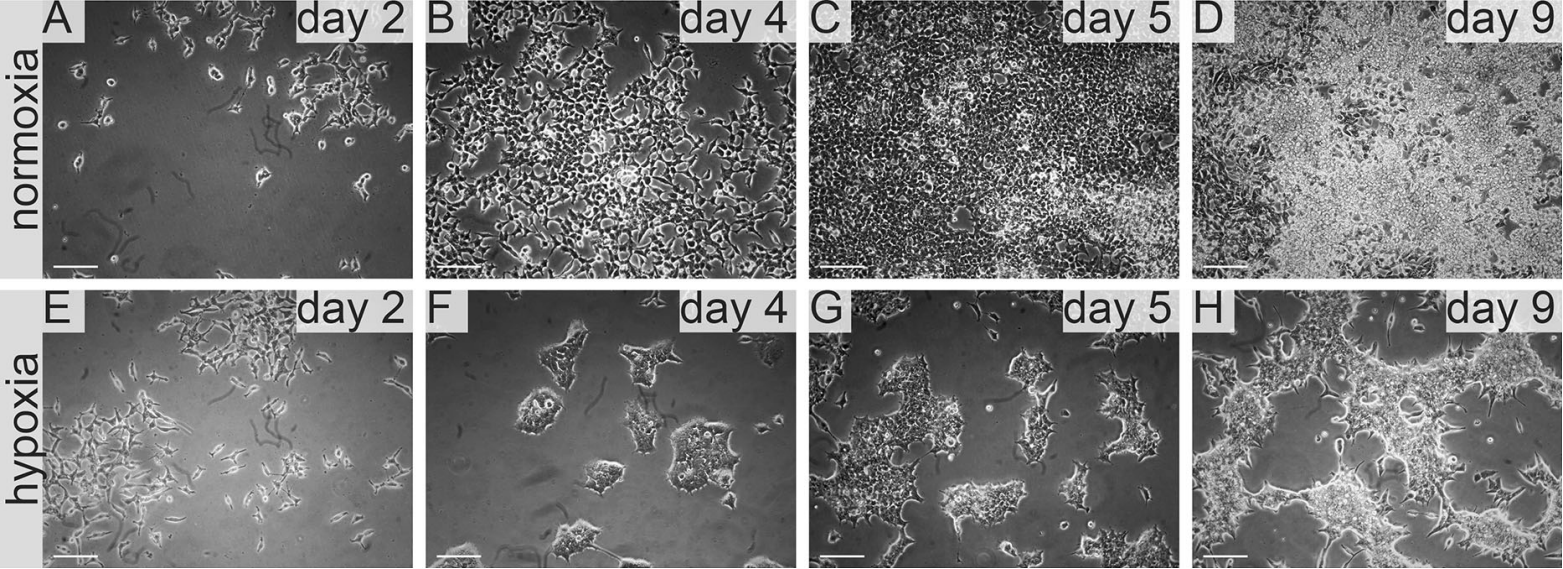
Human kidney cancer stem cell growth in NutriStem medium in normoxia and hypoxia. Representative images present HKCC cells cultured in NutriStem medium in normoxi on **a** day two, **b** day four, **c** day five, **d** day nine; and in hypoxia, on **e** day two, **f** day four, **g** day five, **h** day nine. Scale bar = 100 μm

**Fig. 9:**
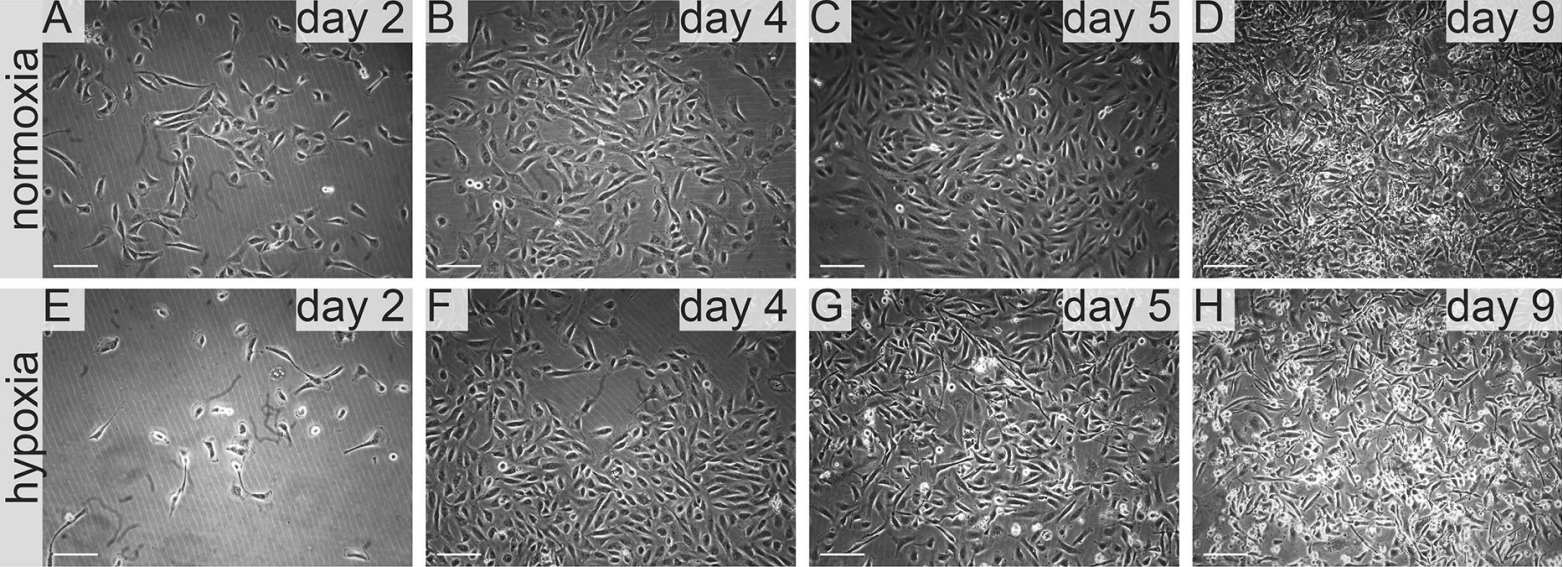
786-0 RCC primary tumour cells in FreeStyle medium in normoxia and hypoxia. Representative images present 786-0 cells cultured in FreeStyle medium in normoxia on **a** day two, **b** day four, **c** day five, **d** day nine; and in hypoxia, on **e** day two, **f** day four, **g** day five, **h** day nine. Scale bar = 100 μm

**Fig. 10:**
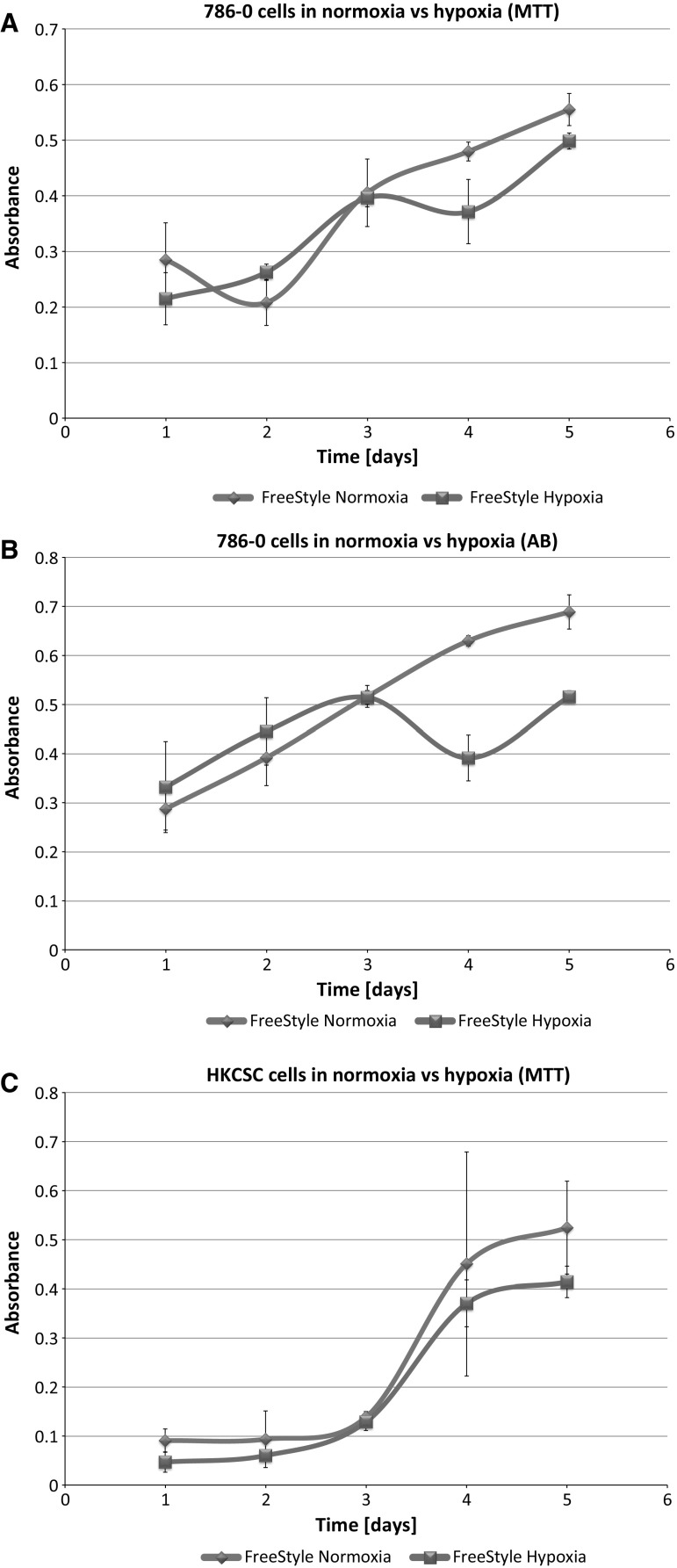

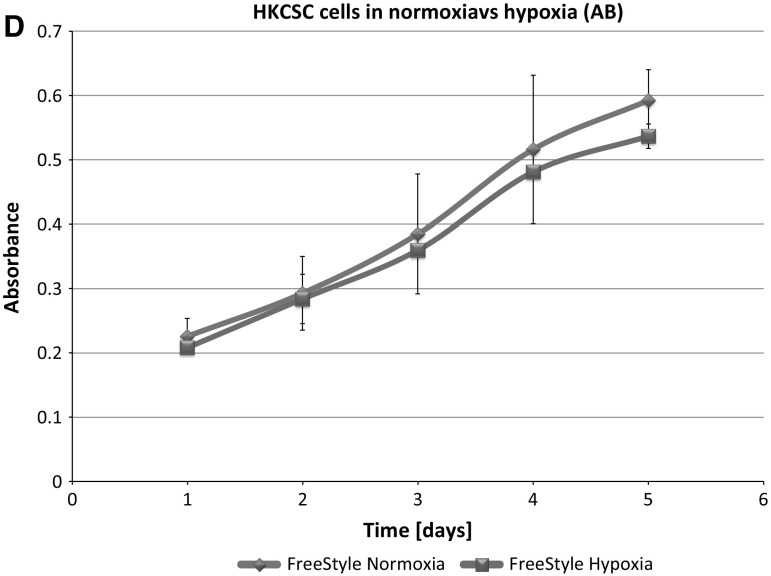
Renal cancer cells viability in FreeStyle medium. 786-0 cells viability in FreeStyle medium in normoxia and hypoxia as determined by MTT (**a**) and Alamar Blue (AB) **b** test; HKCSC cells viability in FreeStyle medium in normoxia and hypoxia as determined by MTT (**c**) and Alamar Blue (AB) **d** test

**NutriStem** medium had a specific effect on 786-0 cell line culture. Initially cells cultured in this medium showed similar proliferation rate in normoxia and hypoxia. Notably proliferation rate was lower than for cultures supplemented with FreeStyle (Figs. 9, 10) or IS293 medium (Figs. 3, 5), however we observed normal cell morphology and no cell detachment until day 5 in both normoxic and hypoxic conditions. In 100% NutriStem medium cells start detaching. On day 9 we observed that over 50% of the cells detached in hypoxia culture, whereas no cell detachment (< 5%) was visible for cells cultured in normoxia. In normoxia 786-0 proliferated at a typical rate in Nutri Stem medium (at 25–100%) and maintained normal morphology (Figs. 7, 8, Table 1). HKCSC proliferated rapidly in NutriStem in normoxia and became confluent on day 5. On the subsequent days HKCSCs overgrew in multiple layers. In hypoxia HKCSCs formed cell clusters in NutriStem medium. The greatest change of cell morphology was observed in this medium for both renal cancer cell types tested.

### FreeStyle medium provided optimal conditions for RCC culture

Interestingly we noticed that 786-0 cells cultured in medium suplemented with **FreeStyle** medium proliferated faster than in 100% control medium both in normoxia and hypoxia. As we gradually changed the control DMEM-FBS medium containing serum to serum-free defined FreeStyle medium we noticed no adverse effects for the cells on either of the steps. In normoxia 786-0 proliferated freely in 25–100% FreeStyle medium. 786-0 did not alter the cell morphology in hypoxia even after 72 h of FreeStyle medium treatment. In hypoxia 786-0 proliferate in 25–100% FreeStyle medium, and were viable with normal morphology. Low number < 5% cells detached (Fig. 9). In FreeStyle medium normoxia slightly promoted cell proliferation (Figs. 4, 10, Table 1).

**Fig. 11:**
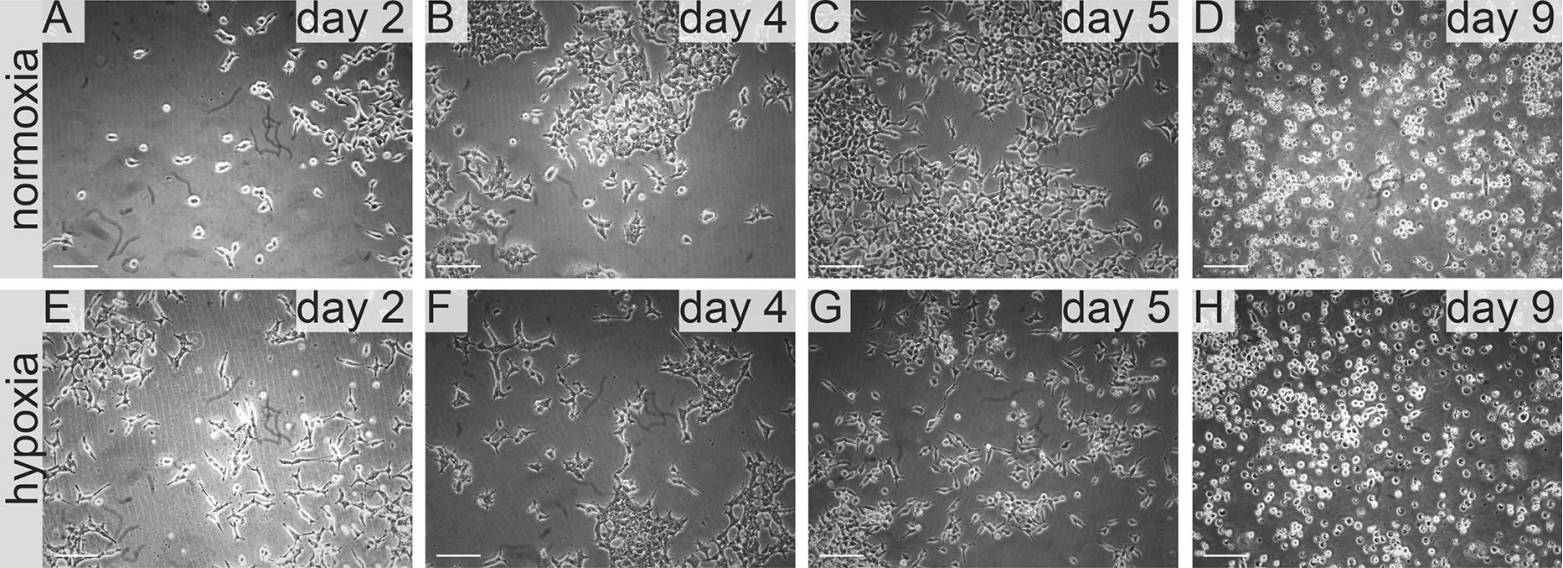
Human kidney cancer stem cells in FreeStyle medium in normoxia and hypoxia. Representative images present HKCC cells cultured in FreeStyle medium in normoxia on **a** day two, **b** day four, **c** day five, **d** day nine; and in hypoxia, on **e** day two, **f** day four, **g** day five, **h** day nine. Scale bar = 100 μm

HKCSC expanded as suspension culture in FreeStyle medium (Figs. 10, 11). Cells in 100% FreeStyle medium proliferated slowlier than in DMEM HG or RPMI medium (Figs. 4, 10), but were able to maintain normal growth over multiple passages. In FreeStyle medium RCC cells grew with average doubling time of 1 day (24 h) to a maximum concentration of up to 3–4 × 10^6^ cells/ml with over 90% viability. Cell morphology of cells remained consistent over time. HKCSCs proliferated in FreeStyle medium in normoxia rapidly, and in 100% FreeStyle high number of cells detached and cells further grew and proliferated in suspension culture. In hypoxia HKCSCs proliferated slowlier than in normoxia and developed viable floating culture in pure FreeStyle medium. HKCSC cells in FreeStyle medium displayed a round lymphoblast-like spherical.

morphology and underwent proliferation in suspension without attaching to the flask surface. We also observed that cells cultured in FreeStyle medium for extended period of time (over 3 weeks) also showed no abnormal morphology or full cell detachment (Fig. 11). RCC cells in FreeStyle medium were viable also after splitting and remained viable up to 27 days in culture (Table 1).

### Adaptation is required for optimal SF-cell culture

**Fig. 12:**
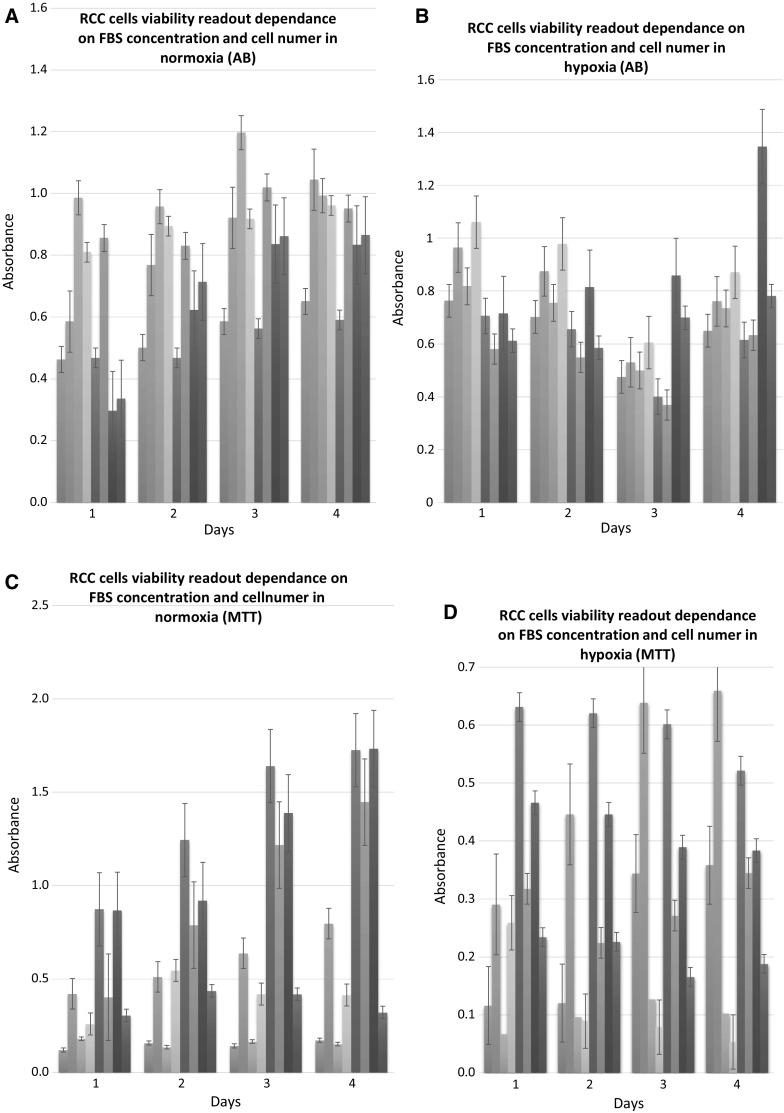
Optimization of RCC cells growth conditions. 786-0 and HKCSCs growth in different media and cell seeding in **a** normoxia (left to right: 786-0 cells cultured in RPMI 10% FBS, seeded 500c/well (*blue*) 786-0 cells cultured in RPMI 10% FBS, seeded 2000c/well (*orange*) HKCSC cells cultured in RPMI 10% FBS, seeded 5000c/well (*grey*) HKCSC cells cultured in RPMI 10% FBS, seeded 2000c/well (*yellow*) 786-0 cells cultured in RPMI 2% FBS, seeded 500c/well (*dark blue*) HKCSC cells cultured in RPMI 2% FBS, seeded 5000c/well (*green*) 786-0 cells cultured in RPMI 2% FBS, seeded 2000c/well (*navy blue*) HKCSC cells cultured in RPMI 2% FBS, seeded 2000c/well (*brown*)) and **b** hypoxia measured by Alamar Blue (AB) (left to right: HKCSC cells cultured in RPMI 2% FBS, seeded 2000c/well (*blue*) HKCSC cells cultured in RPMI 2% FBS, seeded 5000c/well (*orange*) HKCSC cells cultured in RPMI 10% FBS, seeded 2000c/well (*grey*) HKCSC cells cultured in RPMI 10% FBS, seeded 5000c/well (*yellow*) 786-0 cells cultured in RPMI 2% FBS, seeded 2000c/well (*dark blue*) 786-0 cells cultured in RPMI 2% FBS, seeded 500c/well (*green*) 786-0 cells cultured in RPMI 10% FBS, seeded 2000c/well (*navy blue*) 786-0 cells cultured in RPMI 10% FBS, seeded 500c/well (*brown*)) and in **c** normoxia (left to right: 786-0 cells cultured in RPMI 10% FBS, seeded 500c/well (*blue*) 786-0 cells cultured in RPMI 10% FBS, seeded 2000c/well (*orange*) 786-0 cells cultured in RPMI 2% FBS, seeded 500c/well (*grey*) 786-0 cells cultured in RPMI 2% FBS, seeded 2000c/well (*yellow*) HKCSC cells cultured in RPMI 10% FBS, seeded 5000c/well (*dark blue*) HKCSC cells cultured in RPMI 10% FBS, seeded 2000c/well (*green*) HKCSC cells cultured in RPMI 2% FBS, seeded 5000c/well (*navy blue*) HKCSC cells cultured in RPMI 2% FBS, seeded 2000c/well (*brown*)) and **d** hypoxia (left to right: 786-0 cells cultured in RPMI 10% FBS, seeded 500c/well (*blue*) 786-0 cells cultured in RPMI 10% FBS, seeded 2000c/well (*orange*) 786-0 cells cultured in RPMI 2% FBS, seeded 500c/well (*grey*) 786-0 cells cultured in RPMI 2% FBS, seeded 2000c/well (*yellow*) HKCSC cells cultured in RPMI 10% FBS, seeded 5000c/well (*dark blue*) HKCSC cells cultured in RPMI 10% FBS, seeded 2000c/well (*green*) HKCSC cells cultured in RPMI 2% FBS, seeded 5000c/well (*navy blue*) HKCSC cells cultured in RPMI 2% FBS, seeded 2000c/well (*brown*)) measured by MTT test. (Color figure online)

**Fig. 13:**
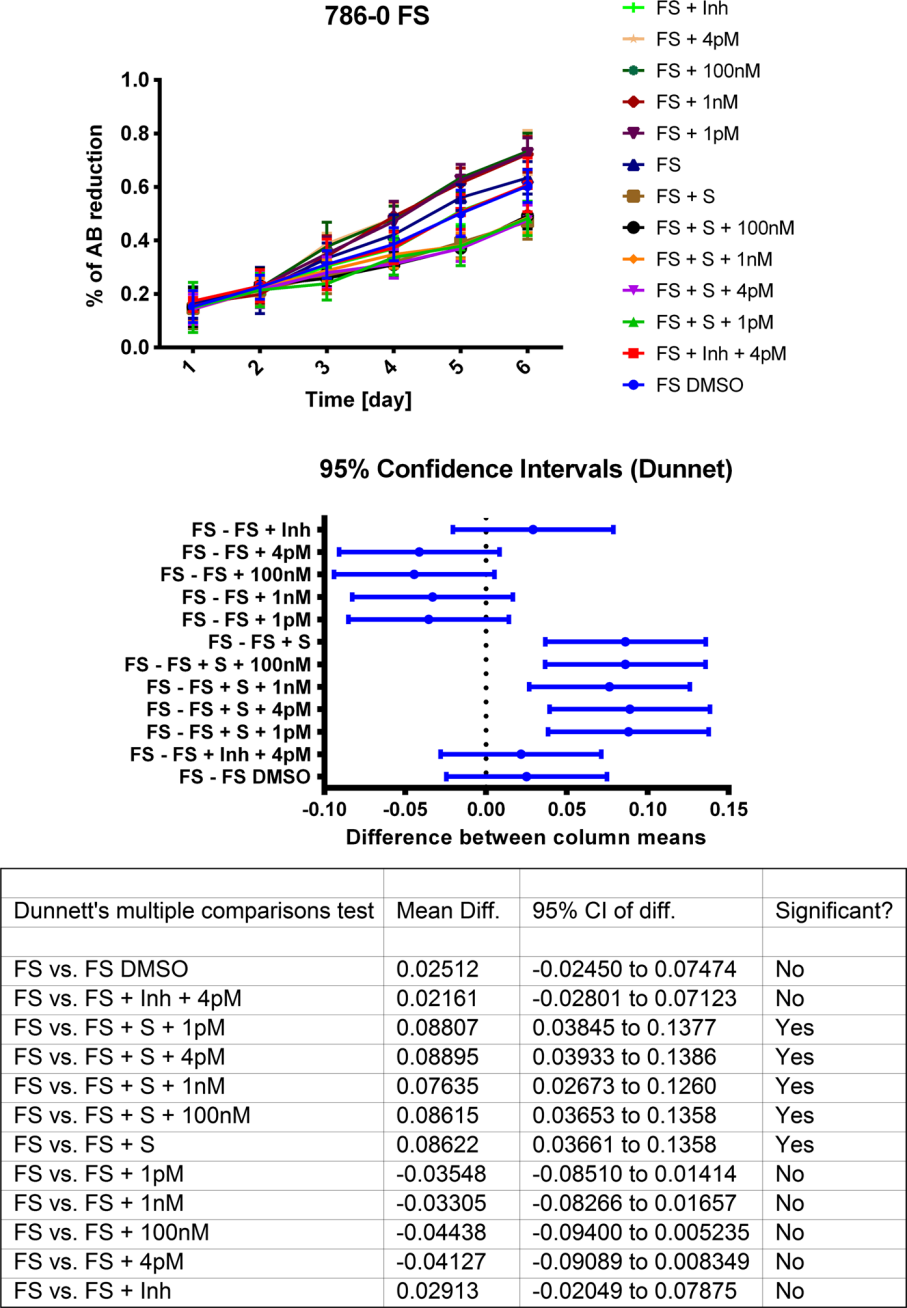
RCC cell growth and hormonal (triiodothyronine) induction in Free Style medium. Presented is percent reduction of Alamar Blue over 6 days. Mean and SD, 95% confidence intervals and statistical comparison two-way ANOVA followed by Dunnett’s multiple comparisons test: FS—Free STyle Medium; Inh—inhibitor of thyroid hormone receptor (antagonist 1-850); S—sunitinib; 4pM-100nM—concentration of triiodothyronine used

Specific adaptation steps are required for optimum serum free-medium performance of RCC culture. Sequential adaptation from FBS-supplemented media should be achieved with gradual adaptation with SF-medium using stepwise reduction from 10% FBS-supplemented medium—with at least ssequential ratios of 1:1, 1:2, 1:4, 0:1 every 24 h after cell attachment (Figs. 3, 5, 6, 7, 8, 9, 11). Subculture of RCC cells from FBS enriched medium to serum-free culture was optimal when performed with 3–5 × 10^5^ viable cells/mL (Fig. 12). Transfer of RCC cells is most effective in their logarithmic phase of growth at > 85% viability before passaging. After final medium adaptation medium had to be exchanged every 72 h until the viable cell density reached 1–2 × 10^6^ cells/mL in T-75 flask. Subculture of RCC cells was optimal with 3–5 × 10^5^ viable cells/mL starting density (Fig. 12). For subsequent experiments maintaining cells in SF medium was feasible for several passages, with sub-culturing no less than once a week. To provide complete physiological adaptation RCC cells had to be cultured for a minimum of 2 passages in SF-medium before further down-stream experiments (Fig. 13). FreeStyle medium may be feasibly used for hormone signalling tests including T3/T4, as well as insulin. Hormones promoted RCC cell proliferation in comparison to growth in non-supplemented medium. It may also be efficiently used for cytotoxic assays, as sunitinib was shown to inhibit RCC cell proliferation in FreeStyle medium, while cells were viable without drug treatment (Fig. 13).

## Discussion

Results from experiments using FBS in cell culture potentially cannot be replicated due to lot-to-lot variability. Although FBS can support the growth of many cell types including RCC, how it does so and which specific factors are important for each individual cell type has not been identified until now. The presence of multiple hormones, growth factors, vitamins and minerals does not allow to evaluate the effect of specific molecules on cell proliferation. Interactions of FBS derived compounds with additional hormones, cytokines, peptides or drug candidates is un-predictable. In order to evaluate whether the substitution of serum is satisfactory without affecting cell growth by the tested media we tested multiple serum free media. Cell growth was studied up-to one week to provide long term observation period. In the screening of animal derived component free media—three xeno-free defined media and stem cell dedicated media were tested, but only one—FreeStyle medium—significantly enhanced 2D growth of RCC cells and enabled to maintain their epithelial morphology. Moreover FreeStyle medium supported suspension growth of HKCSC cells and promoted round lymphoblast-like spherical morphology, as expected for these stem cells. Previous studies on cancer cell lines have also shown that correlations between in vitro 3D cell culture properties including cell aggregate morphology in suspension culture or hanging drop culture and in vivo cell invasiveness may be found. Growth in stellate, grape-like, mass, or round morphological structure arises as different gene expression activation pattern (Kenny et al. 2007).

HEK cells were often used as renal cell culture model and therefore HEK-dedicated media were tested. The original HEK293 (CRL-1573) cells were derived in 1973 from the kidney of an aborted human embryo of unknown parenthood and transformation with Adenovirus 5 DNA and selection of a single transformed clone (Graham et al. 1977). HEK cells are traditionally cultured in Minimum Essential Medium supplemented with 10% FCS and 1 mM sodium pyruvate. Optimal concentrations of supplements in HEK 293 cell culture medium were previously defined as 19.8 mg/L of r-insulin, 1.6 mg/L of r-transferrin and 0.9X of the lipid mix. In the presence of this optimal combination and concentrations of supplements HEK 293 cells reach a maximum cell density of 5.4 × 10^6^ cells/mL. For this cell line un-supplemented Freestyle medium supports HEK293 cell growth up 3 × 10^6^ cells/mL (Cervera et al. 2011). In our study Free Style is a complete medium that supported growth of RCC cells without further supplementation. It provided serum-free and low protein culture conditions.

In renal cell studies other FBS-free media have been described before. Madin-Darby canine kidney (MDCK) cells are able to grow in synthetic medium supplemented with five factors only: insulin, transferrin, triiodothyronine, hydrocortisone, and prostaglandin E1. In the absence of FBS these five factors enabled cell proliferation of renal cells up-to 1 month. In these culture conditions, growth rate of MDCK was not slower than in serum-supplemented medium (Taub et al. 1979). RCC 786-0 cells were recently shown to be able to proliferate and express transfected genes in Opti-MEM^®^ (Reduced-Serum Medium). This is an improved Minimal Essential Medium (MEM) that allows for a reduction of FBS supplementation by at least 50% with no change in cell growth rate or morphology and it was primarily tested for HEK cells (Wang et al. 2015). Moreover 786-0 RCC cells were able to survive in FCS-free Roswell Park Memorial Institute Medium (RPMI-1640; Sigma-Aldrich) supplemented with 2 mM l-glutamine (Lonza), 1% sodium pyruvate (GIBCO), 1% non-essential amino-acid mixture (GIBCO), and 1% penicillin/streptomycin (PAA) up-tp 72 h (Stehle et al. 2015). Mechanism enabling RCC cell survival in serum free conditions had been investigated. Constitutive HIF activation was shown as required for serum-independent growth of 786-0 VHL^−/−^ RCC cells in culture, as well as for tumour formation in xenograft nude mice by these cells. In the absence of exogenous growth factors or serum in culture medium, VHL-deficient cells were able for autonomous growth and rapid proliferation as a consequence of pathological HIF activation (Smith et al. 2005). In SF medium composed of DMEM supplemented with 1% insulin-transferrin-selenium (ITS; Invitrogen, Burlington, Ontario, Canada) silencing of ADAM17 was sufficient to abrogate the ability of both 786-0 cells to proliferate to levels similar to those observed in renal carcinoma cells upon stable silencing of EGFR. After 786-0 cells have been cultured for 72 h in the absence of serum, addition of exogenous TGF-α activated EGFR signalling and reinitiated cell proliferation. ADAM17-mediated processing of pro-TGF-α into its soluble form is essential for EGFR phosphorylation and critical for the establishment of the TGF-α/EGFR autonomous proliferation of RCC (Franovic et al. 2006).

Cells growing in serum-free conditions are more susceptible to changes in pH and toxic substances. It is also important to point out that while the low glucose conditions (DMEM low glucose) provided glucose at levels approximating that of blood (1 g/L or ~ 5 mM), whereas the *in* *vitro* conditions do not exactly reflect those of the *in* *vivo* situation, as medium in culture is exchanged once in 24–72 h, while blood in vivo distribute glucose at all times. Moreover it is also possible that the nutrients in the cell culture media are rapidly used up in low glucose conditions, but not in DMEM HG. The exact kinetics of glucose metabolism and resultant RCC cell viability decline should be monitored (Farrell et al. 2015). In serum free and low nutrient conditions frequent cell viability re-analysis is recommended. MTT test measures viability, proliferation and activation of cells by monitoring capacity of cellular mitochondrial dehydrogenase enzyme in living cells that reduce yellow water-soluble substrate MTT into insoluble dark blue to purple formazan. The amount of formazan produced is directly proportional to the cell number. The MTT assay has greater applicability in detection of cells which are not dividing but are still metabolically active. It can therefore be used to distinguish between proliferation and cell activation (Patel et al. 2013). On the other hand MTT assay may suggest higher viability and give rise to interpretation of relatively lower inhibition by cytotoxic drugs than the ATP assay (Ulukaya et al. 2008). MTT is reduced by FMNH, FADH, NADH, NADPH, but not by cytochromes. On the contrary Alamar Blue is reduced by cytochromes, FMNH, FADH, NADH, and NADPH, while MTT will be reduced by FMNH, FADH, NADH, NADPH, but will not be reduced by cytochromes. At the same time it must be remembered that Alamar Blue assay is sensitive to protein concentration in culture media and therefore SF-media represent reliable culture method to monitor cells with resazurin (Goegan et al. 1995). Also accumulation of the fluorescent product of Alamar Blue in the medium could lead to an overestimation of cell population (O’Brien et al. 2000).

## Conclusions

The main goal of the present study was to provide different RCC cell culture platforms which are suitable for a wide range of gene expression applications including analysis of pathway activation dependent on hormones or growth factors, including endo-, para- and autocrine studies. From the range of applications in which HEK293 media may be used, the work carried out in this project was directed towards endocrine oncology.

Expanding RCC cells under serum free conditions enable to develop more controlled and defined biomimic cell culture system, as needed for down-stream applications. Proliferation of RCC cells in serum free conditions allow researchers to develop easily controlled and defined biomimic cell culture system. Such biomimic model is required for preclinical academic projects, to have control over all culture variables in cell line based experiment, for development of hypothesis-driven results and for candidate drug testing in cell culture. Incubation of mammalian cells in serum free medium is required to avoid interference from FBS contaminants affecting gene expression and cell secretome profile. In addition, the duration of incubation in serum-free medium can influence cell secretome profile. Cell culture must be individually optimized as in sub-optimal xeno-free medium increased cell death may be reported, as well as increased release of intracellular proteins (Stehle et al. 2015).
